# Trigeminal artery anatomical aspects

**DOI:** 10.1007/s00276-024-03553-0

**Published:** 2025-01-04

**Authors:** Ionuț Bulbuc, Dan Marcel Iliescu, Constantin Dina, Constantin Ionescu, Petru Bordei

**Affiliations:** Ovidius” University From Constanţa, Constanța, Romania

**Keywords:** Trigeminal artery, Anatomic aspects

## Abstract

**Purpose and background:**

The trigeminal artery is a rare anatomical variant, representing an embryonic vestige of the anastomosis between the internal carotid artery and the posterior circulator system, that can be asymptomatic or could have vast clinical manifestations produced by insufficient flow or by vascular nervous conflicts. This study is an anatomical presentation of 3 trigeminal artery cases observed at Medimar Imagistic Services Constanta.

**Methods:**

The 3 trigeminal artery cases were discovered on a 860 magnetic resonance angiographies (0.35% of cases), made on a GE HD/e 8ch 1.5 T.

**Results:**

In all 3 cases, the arteries were rising from the right internal carotid artery and one from the left internal carotid artery, in 2 cases the origin was on the superior surface, and in one case, on the anterolateral surface, in all 3 cases from C4 horizontal cavernous segment. The artery caliber was between 2.7 mm and 5.1 mm; the artery length was between 26 and 32 mm. Other associated vascular malformations were: partial or total basilar artery hypoplasia, in one case posterior communicant artery agenesis, contralateral vertebral artery hypoplasia, posterior cerebral artery hypoplasia (in 2 cases), and no anastomosis between the P1 segment of the posterior cerebral artery with the posterior communicating artery (one case).

**Conclusions:**

While the trigeminal artery is a rare anatomical variant, it’s still a very important vessel situated in the posterior cerebral fossa, which needs to be taken into account in the case of neurosurgical interventions.

## Introduction

Trigeminal artery is a primitive anastomosis of anterior carotidian arterial system with the posterior vertebrobasilar. Those anastomoses are going parallel with cranial nerves except proatlantal artery that is extra cranial. The persistence of those anastomoses regarding frequency, in decreasing order, is: trigeminal artery, hypoglossal artery, otic artery. Those anatomical variations could be understood by following the stages of embryonic cerebral circulation development. Cerebral arteries are developing synchronously with encephalic structures in direct relation with their stadial changes. Padget [13, 14] have made a study, in 1948, about cerebral arterial system development in embryo, especially ophthalmic artery, stapedial artery and trigeminal artery.

The exact order of cranial vessels is varying from one individual to another. After [13, 14] study, the development has seven successive phases.

In first phase (28 days to 32 days, postovulatory age), first and second aortic arches start to involute. The pair aortic segments are extending cranially and form the third arch, and so, the primitive carotid artery, on each side. This artery seems to rise from a vascular plexus and is dividing distally in two branches: primitive trigeminal artery and a cerebral artery that is going to Rathke pouch where is anastomosing with the opposite one. Carotid artery will form two vascular compartments: one rostral that is ending in olfactory area, and one caudal that is ending in an mesencephalon plexus. Basilar artery is not yet formed. Instead, paired longitudinal neural arteries are bordering the posterior brain, on each side and are connecting laterally with the primitive posterior brain plexuses. Neural arteries are receiving blood flow from two sources: trigeminal artery, cranially and firs segmental cervical artery caudally [3].

Phase 2. Carnegie stage 14 (maximum length 5 mm to 7 mm; 32 ± 1 days postovulatory age). Internal carotid artery is well defined. Basilar artery it starts to form. Caudal part of each internal carotid artery is developing a second anastomosis with cranial, terminal part of ipsilateral neural arteries that is the posterior communicating artery. This new artery will replace, in short time, trigeminal artery and will be a major flow source for longitudinal neural artery (future basilar artery).

In next stages it will be forming progressively, vertebral arteries, superior cerebellar arteries, choroid arteries, posterior cerebral arteries, anterior cerebral circulator system, that in phase 7, at 9 weeks postovulatory age, cerebral arteries, to have the adult shape [7, 14].

Suttner [18] described trigeminal artery, on cadaveric specimens studies, with origin on the superomedial surface of distal 1/3 internal carotid artery intracavernous horizontal segment. From it’s origin, goes medial then posteroinferior, passes between internal carotid artery posterior curvature, medially, and hypophysis, laterally, crosses cavernous sinus, being superior and inferior to oculomotor, trochlear and abducens nerves and in most of the cases medial to trigeminal ophthalmic branch. If trigeminal artery has its origin on the posteromedial internal carotid artery intracavernous sinus segment, it becomes, extradural at dorsum sellae, medial to abducens nerve. From trigeminal artery can rise inferior hypophyseal artery, posterior meningeal artery and branches for trigeminal nerve [18]. Salas [16] propose a classification of trigeminal arteries on the basis of anatomical relations that it establishes and divides them in a medial type, sphenoidal, in which trigeminal artery passes thru sella turcica, pierces dura and passes lateral from clivus and a lateral type, petrous, in which trigeminal artery follows trigeminal nerve sensory branches and lives Meckel cavum inferior to petroclinoid ligament. Last type seems to be the most encountered one [16].

**Fig. 1:**
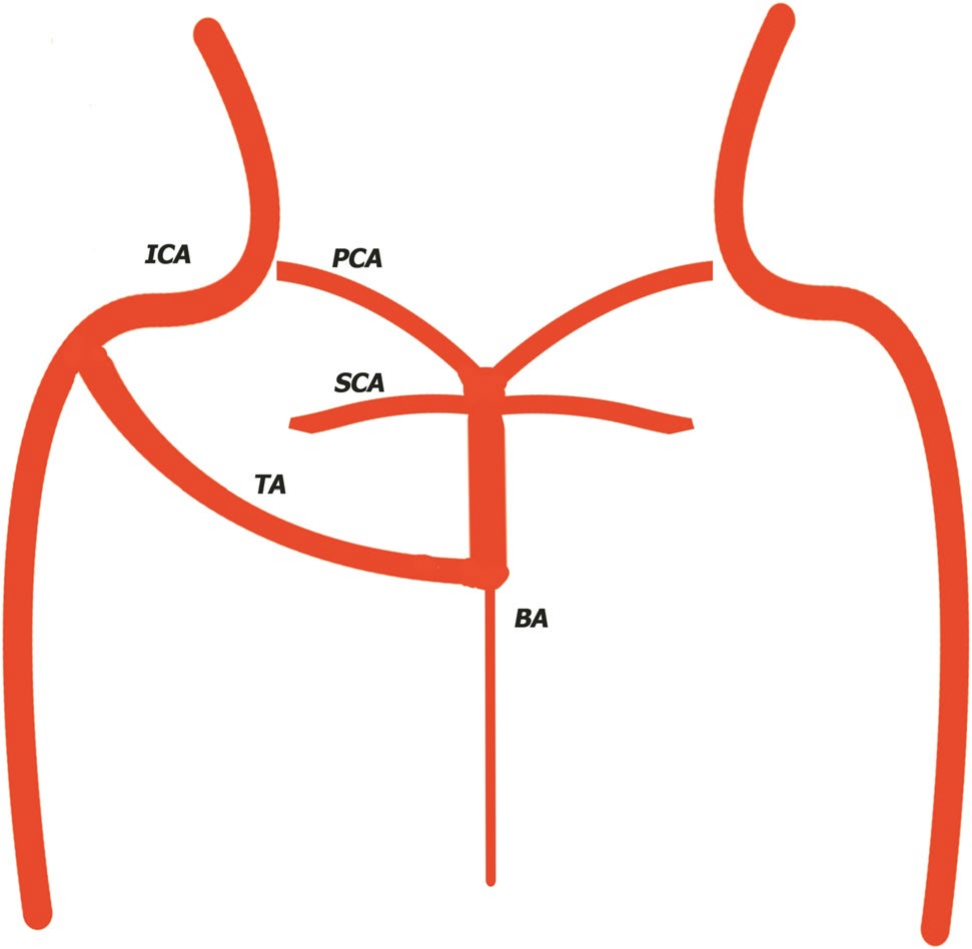
Type 1 Saltzman trigeminal artery. *ICA*- Internal Cerebral Artery, *BA* Basilary Artery, *TA* Trigeminal Artery, *SCA* Superior Cerebellar Artery, *PCA* posterior cerebral artery

Saltzman [17], in studies made on cerebral angiographies, divided in 3 types trigeminal arteries, on the basis of the relation with posterior communicating artery and vascularization territory. Type I (Fig. 1) represent the main flow source for distal part of basilar artery, superior cerebellar arteries, posterior cerebral arteries and associates basilar artery hypoplasia below trigeminal artery anastomosis and ipsilateral posterior communicating artery agenesis.

**Fig. 2:**
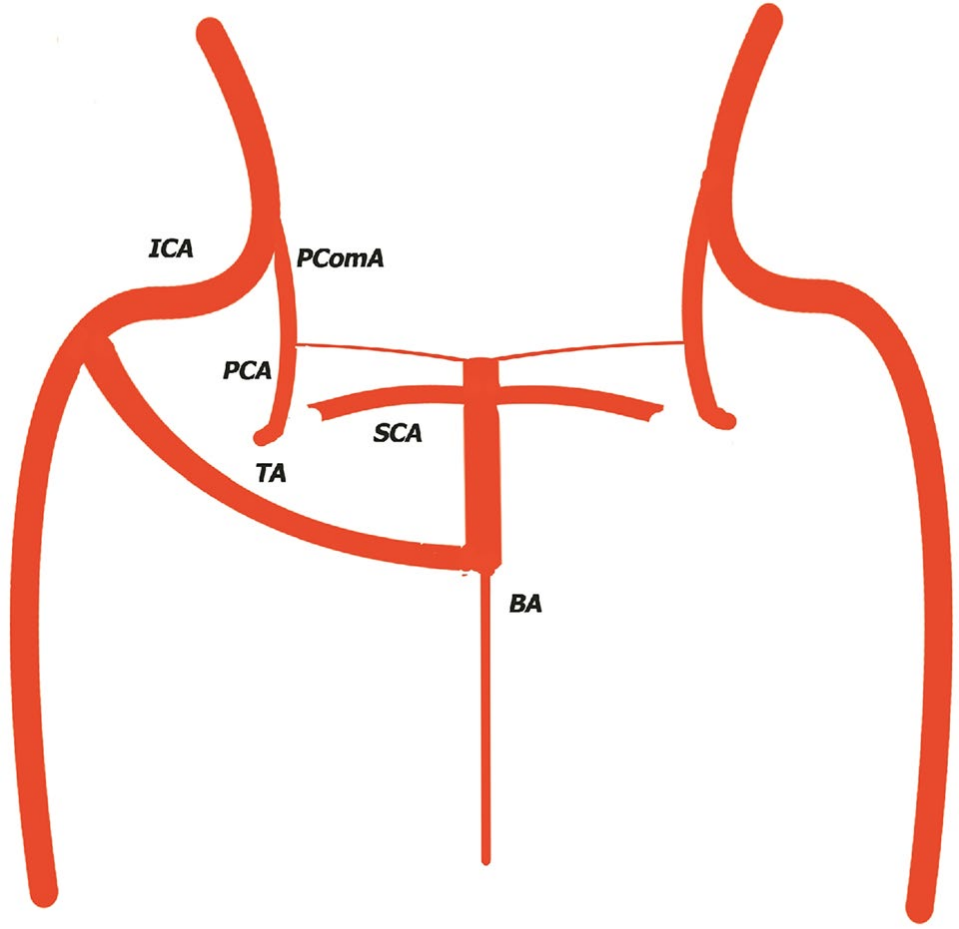
Type 2 Saltzman Trigeminal Artery. *ICA* Internal Cerebral Artery, *BA* Basilary Artery, *TA* Trigeminal Artery, *SCA* Superior Cerebellar Artery, *PCA* posterior cerebral artery, PComA Posterior Communicating Artery

In type II, (Fig. 2) described by [17], trigeminal artery give rise only to superior cerebellar arteries, posterior cerebral arteries, have they origin from posterior communicating arteries, with agenesis/hypoplasia of posterior cerebral artery P1 segment. The basilary artery is hypoplastic below the anastomosis with trigeminal artery.

**Fig. 3:**
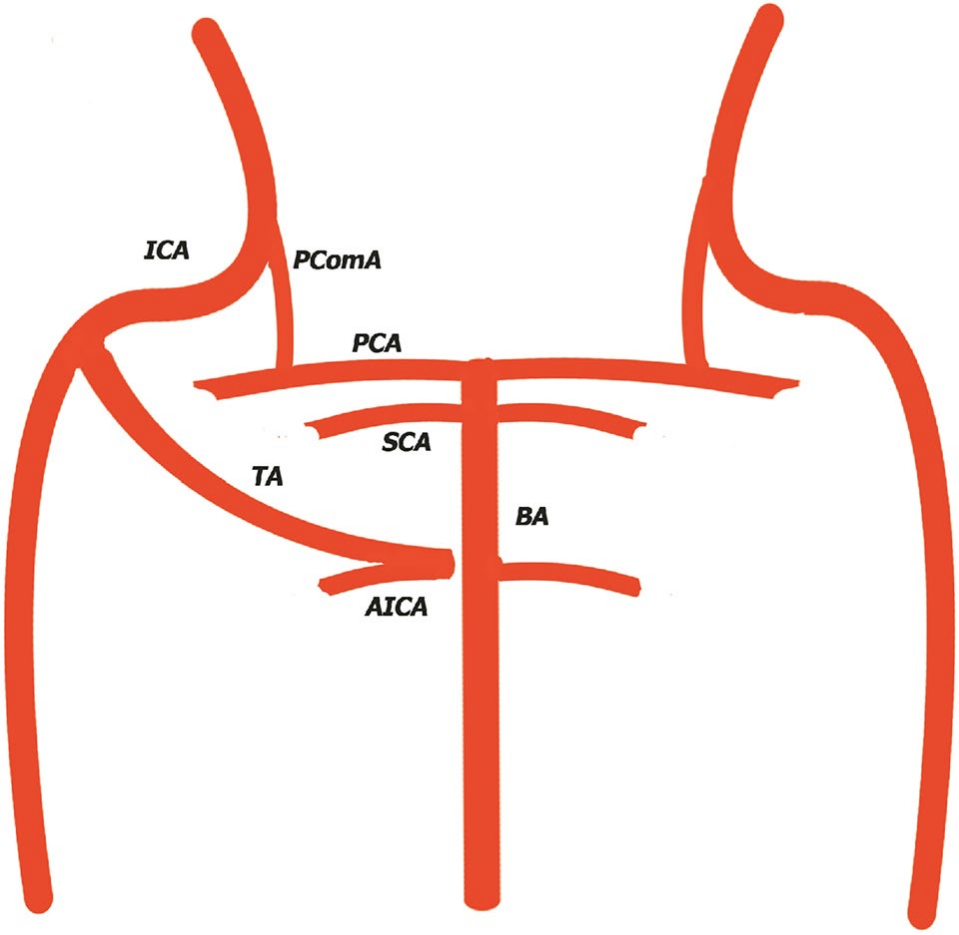
Type 2 Saltzman Trigeminal Artery. ICA- Internal Cerebral Artery, *BA* Basilary Artery, *TA* Trigeminal Artery, *SCA* Superior Cerebellar Artery, *PCA* posterior cerebral artery, *PComA* Posterior Communicating Artery, *AICA* Anterior Inferior Cerebellar Artery

In type III, (Fig. 3), described by Saltzman, trigeminal artery joins with a longitudinal neural artery remnant and gives rise to one cerebellar artery, usually the ipsilateral anteroinferior, not joining the basilar artery [17].

In Saltzman study, the described types of trigeminal arteries prevalence is: 25% type I, 16% type II, 60% type III [17].

In most of the cases, trigeminal artery associates other anatomical variants: ipsilateral posterior communicating artery agenesis or hypoplasia, vertebral artery or basilar artery agenesis or hypoplasia. In 75% of cases is associated with various grades of basilar artery hypoplasia, in this situation, the basilar artery blood flow is assured by the internal carotid artery for brain stem upper part, cerebellum, and same side brain hemisphere [1].

Different studies presented different prevalence for trigeminal artery, most probable due to variable work methods. In it’s study, made on 481  conventional angiographies, Allen [2], sustain an incidence of 0.1% [2]. Chen [5] in a 4650 MRI angiographies study, found 25 cases (0.54%), Uchino [19] with the same method of employment found an incidence of 0.51% [19]. O’uchi [12] in a study made on 16,415 MRI angiographies describes 48 trigeminal arteries (0.29%) and 50 cases of trigeminal artery variants, corresponding to Saltzman type III. Rhee in a 1250 conventional angiographies study, found a incidence of 0.32% [1, 15].

## Material and methods

The study was employed on 860 magnetic resonance angiographies, obtained with GE HD/e 8ch 1.5 T machine at Medimar Imagistic Services Constanta. We used a time of flight sequence with 3D reconstruction technique. To detail vasculonervous relation in cavernous sinus and prepontine cistern we made high resolution T2 sequences. All images were evaluated with a GE workstation, using source images and also maximum intensity projections.

## Results

**Fig. 4:**
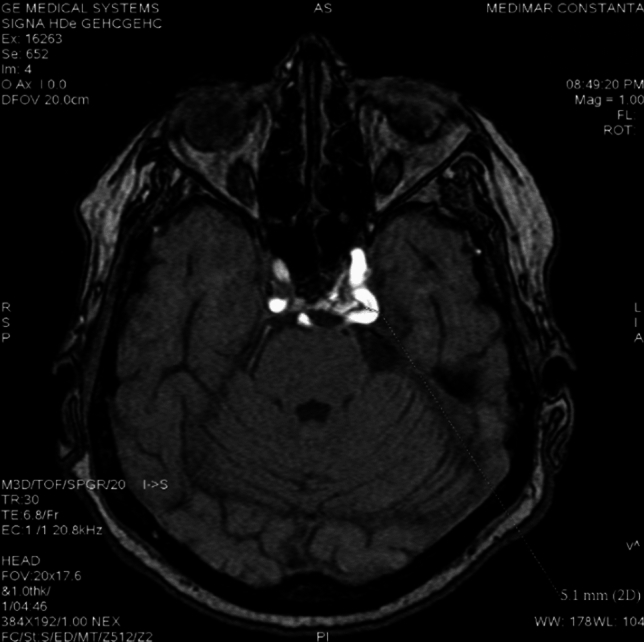
TOF sequence

**Case 1**. A 79 years old female patient, that presented to neurologist for a persistent vestibular syndrome. At MRI exam was discovered a trigeminal artery that originates from the superior surface of proximal right internal carotid artery C4 segment, situated in the cavernous sinus lateral wall. From it’s origin, trigeminal artery is directed superior and lateral, situated inferior to oculomotor and trochlear nerves, medial to trigeminal nerve ophthalmic branch (Fig. 4).

**Fig. 5:**
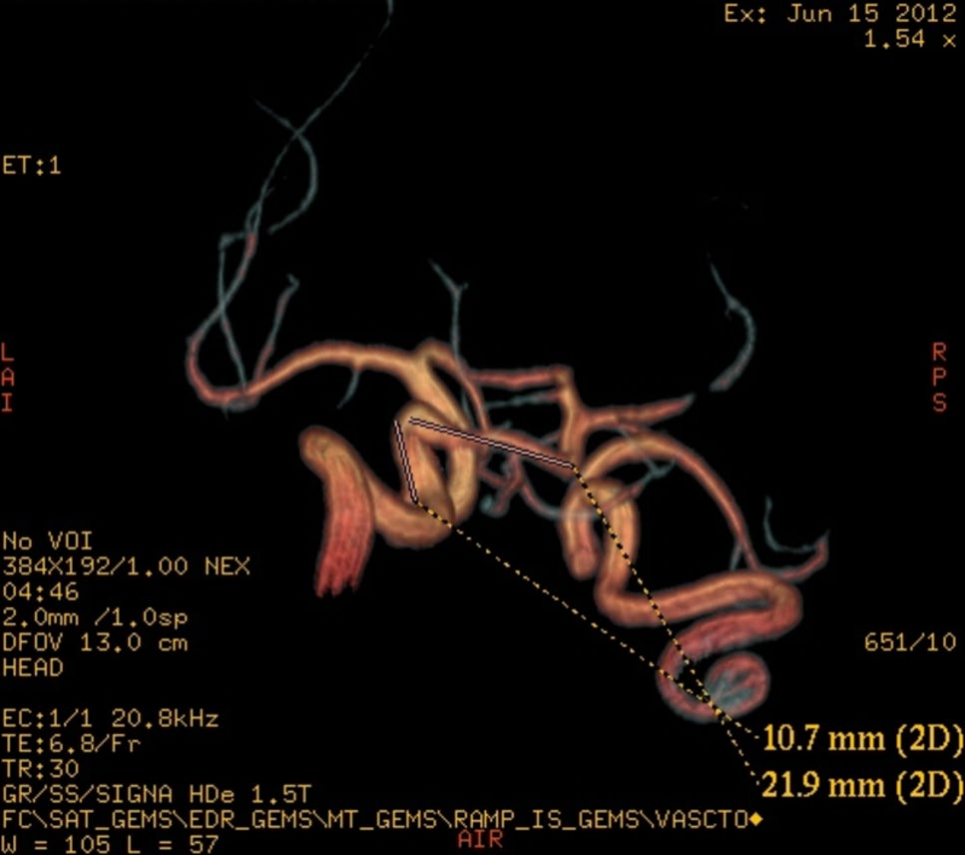
VR reconstruction with length measurements

Anterior to petrous part of temporal bone, the artery is bending in an 90° angle, comes in close relation with trigeminal ganglia, then becomes horizontal, directs posterior and medial, it travers Meckel cavum, pierce dura mater inferior to petroclinoid ligament, medial to trigeminal nerve sensory root and goes in prepontine cistern, medial to trigeminal nerve, superior from abducens nerve, and tangent to pons right half. Trigeminal artery is anastomosing with basilar artery inferior from superior cerebellar artery origin superior to anteroinferior cerebellar arteries origin. The trigeminal artery has a 5.1 mm caliber an a length of 32 mm (Fig. 5).

**Fig. 6:**
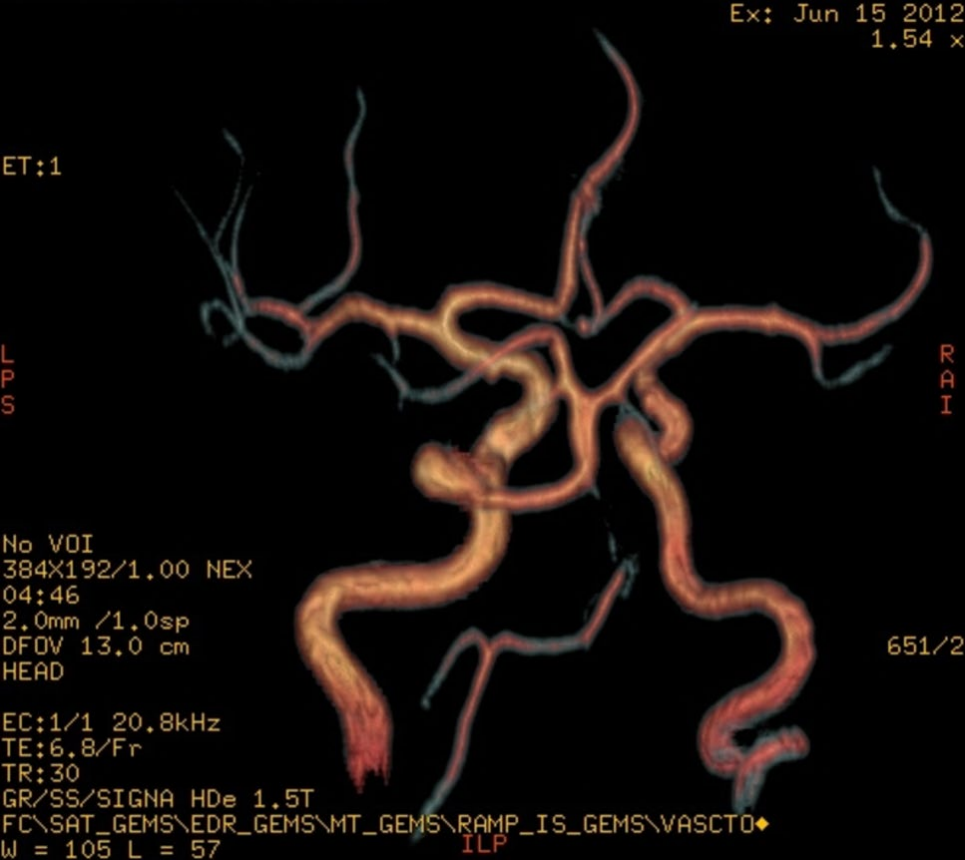
VR reconstruction, of Saltzman type 1 trigeminal artery and contralateral vertebral artery hypoplasia

**Fig. 7:**
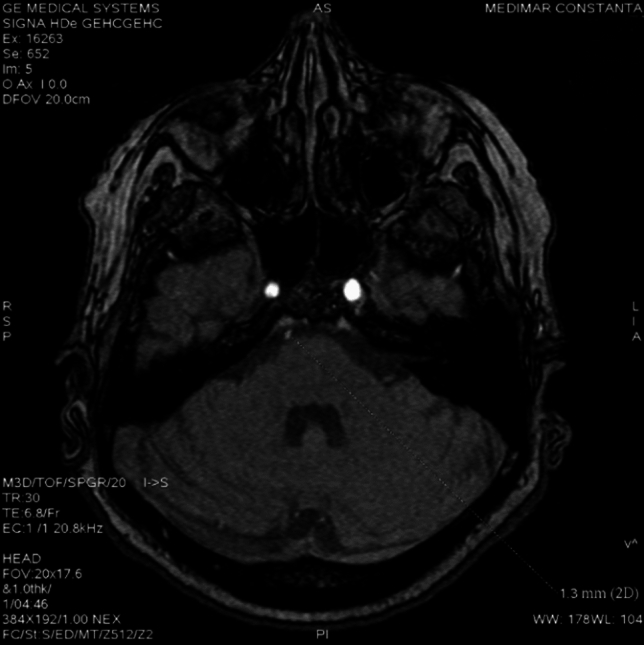
TOF sequence demonstrating  basilar artery hypoplasia

**Fig. 8:**
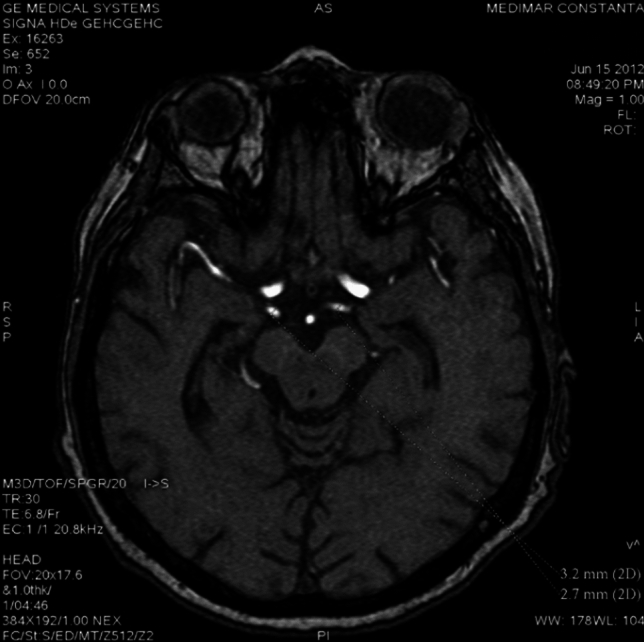
TOF sequence with normal caliber basilar artery above trigeminală artery anastomosis

Trigeminal artery is associated with other anatomical vascular anomalies: basilar artery hypoplasia below anastomosis with trigeminal artery, where it has a caliber of 1.3 mm, superior to anastomosis with a caliber of 4.3 mm. Same side communicating artery cannot be seeing, agenesis, with normal developed posterior cerebral arteries that have a 2.7 caliber on left side, 3.2 mm on right side, so it can be included in Saltzman type I (Fig. 6). Also associates contralateral vertebral artery hypoplasia (Fig. 7*, *Fig. 8).

**Fig. 9:**
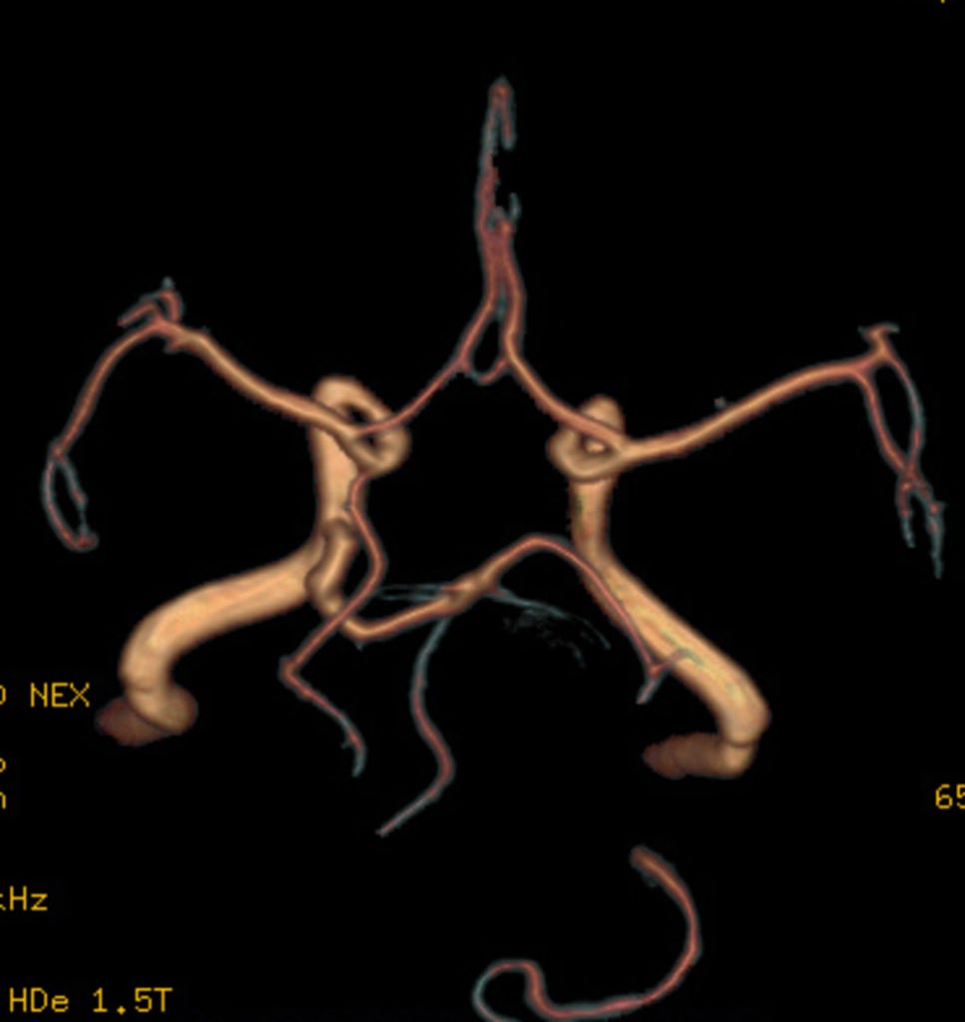
VR reconstruction demonstrating trigeminal artery

**Fig. 10:**
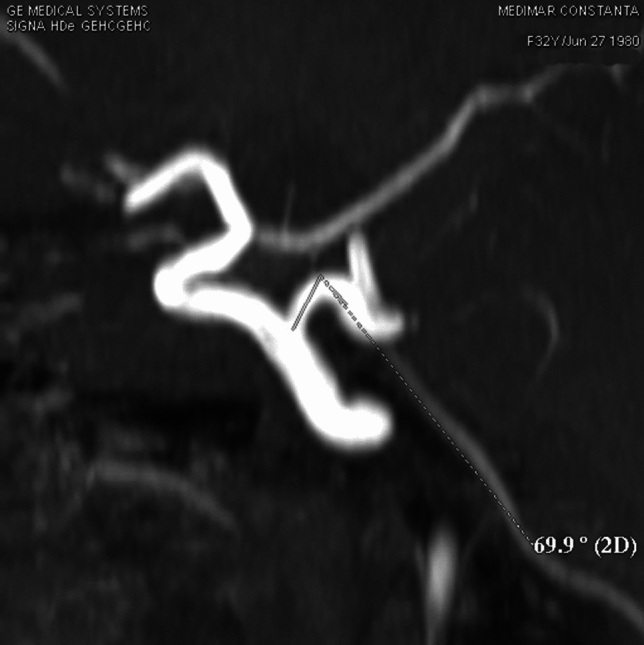
.Angle mesurent in TOF séquence

**Case 2**: A 32 years old female patient that presented to neurologist for left hemicranias. On MRI exam we discovered a trigeminal artery with its origin on left internal carotid artery superior surface in proximal C4 segment. The artery has a short ascending and lateral direction in the cavernous sinus lateral wall, with a length of 6 mm it angles at 70^o^, change its trajectory inferiorly and medially towards Meckel cavum, in this segment is positioned inferior and lateral to oculomotor nerve, medial to trigeminal nerve ophthalmic branch, that it also slightly deviates. The trigeminal artery perforates dura inferior to petroclinoid ligament it becomes horizontal forming an 110^o^ angle and goes medial in prepontine cistern, comes in contact with trigeminal nerve laterally. The artery it’s anastomosing with basilar artery inferior from superior cerebellar artery origin. Trigeminal artery has a total length of 26 mm with a 3.9 mm diameter. From trigeminal artery rises left anteroinferior cerebellar artery at 9 mm distance from the anastomosis with basilar artery (Fig. 9*, *Fig. 10).

**Fig. 11:**
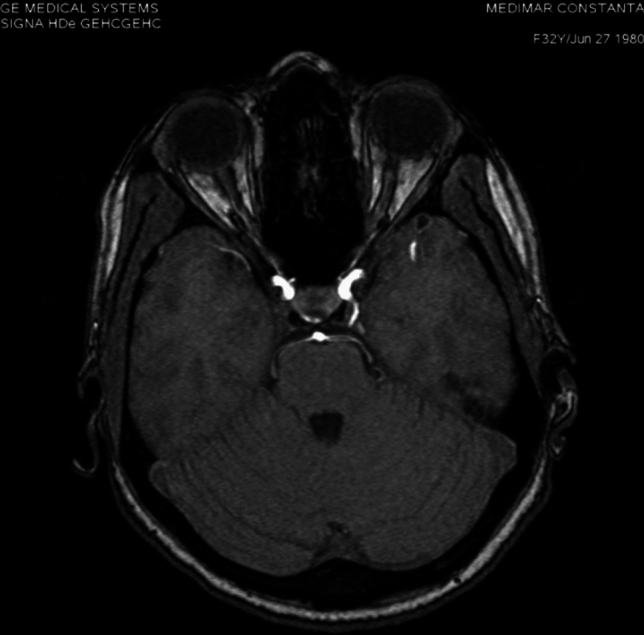
TOF sequence, the developed left posterior communicating artery

**Fig. 12:**
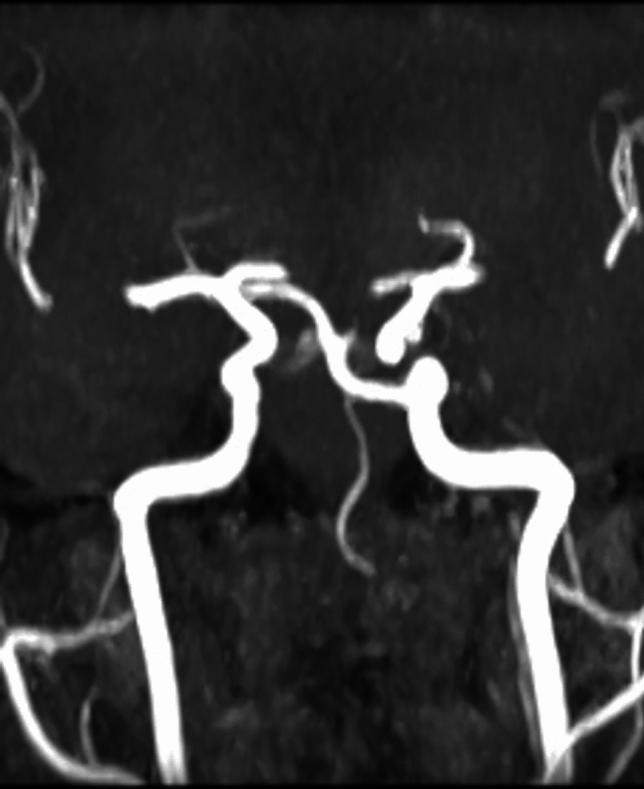
MIP of a TPF sequence demonstrating basilar artery  hypoplasia inferior to trigeminal artery

Trigeminal artery presence it’s associated with other vascular anomalies like: basilar artery hypoplasia, with a 1.8 mm caliber inferior to trigeminal artery anastomosis, 3.4 mm diameter superior from it, posterior cerebral artery hypoplasia that is not anastomosing with ipsilateral posterior communicating artery, it gives only the deep branches and posteromedial choroid artery. Left posterior communicating artery has a 2.1 mm diameter and is distributing in posterior cerebral artery cerebral vascular territory (Fig. 11). Right cerebral artery is normal developed with a 2.4 mm diameter. Trigeminal artery is also associated with right vertebral artery hypoplasia (Fig. 12). In relation with Saltzman classification, on the base of vascular territories, this trigeminal artery is an intermediary type with ipsilateral posterior cerebral artery hypoplasia, specific to type II and normal developed right posterior cerebral artery associated with basilar artery hypoplasia specific to type I.

**Fig.13:**
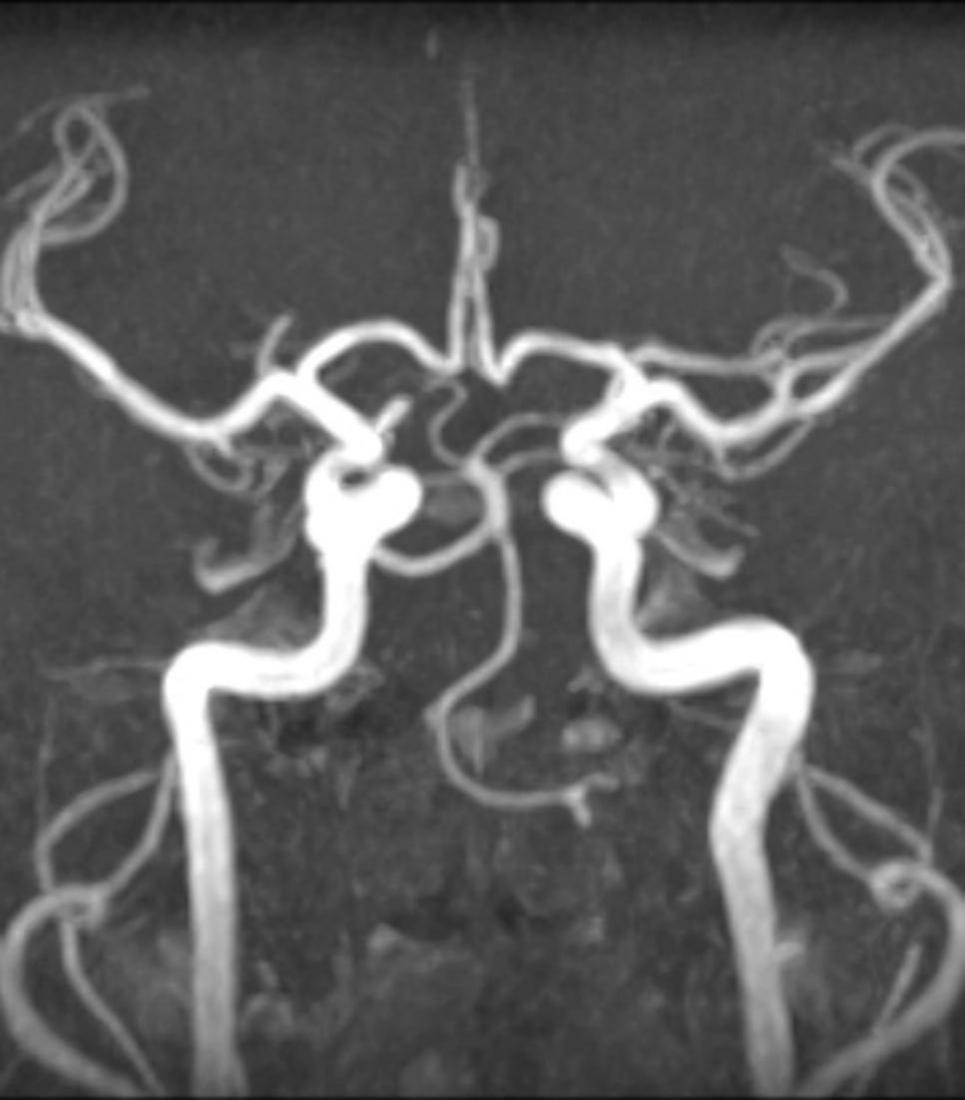
Coronal MIP of a TOF sequence  with right trigeminal artery

**Fig. 14:**
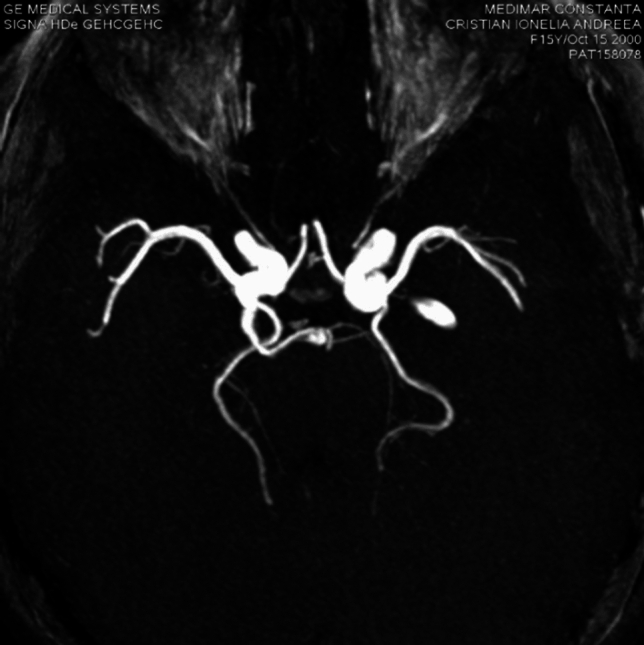
Axial MIP of TOF sequence of trigeminal artery anastomosing inferior to superior cerebellar artery

**Case 3**: 16 years old female patient presents to neurologist for hemicranias and diplopia, with right eye abduction deficiency. On MRI exam we discovered right rectus lateralis muscle edema and a trigeminal artery from right internal carotid artery. The origin of this artery is on C4 segment superolateral surface, it has a sinous aspect, it form al loop with lateral convexity at 18^o^, descends in cavernous sinus, medial to trigeminal nerve ophthalmic branch, comes in direct contact with abducens nerve at it entrance into cavernous sinus (Fig. 13), is positioned inferior to oculomotor nerve, angulates at 700 turn posteriorly and medially, it traverse Meckel cavum medial and inferior to trigeminal ganglia, pierces dura and change direction at 90^o^, becoming horizontal in prepontine cistern where it’s anastomosing with basilar artery inferior to superior cerebellar artery origin (Fig. 14). The trigeminal artery, in this case, has a 2.7 mm diameter and 32 mm length.

**Fig. 15:**
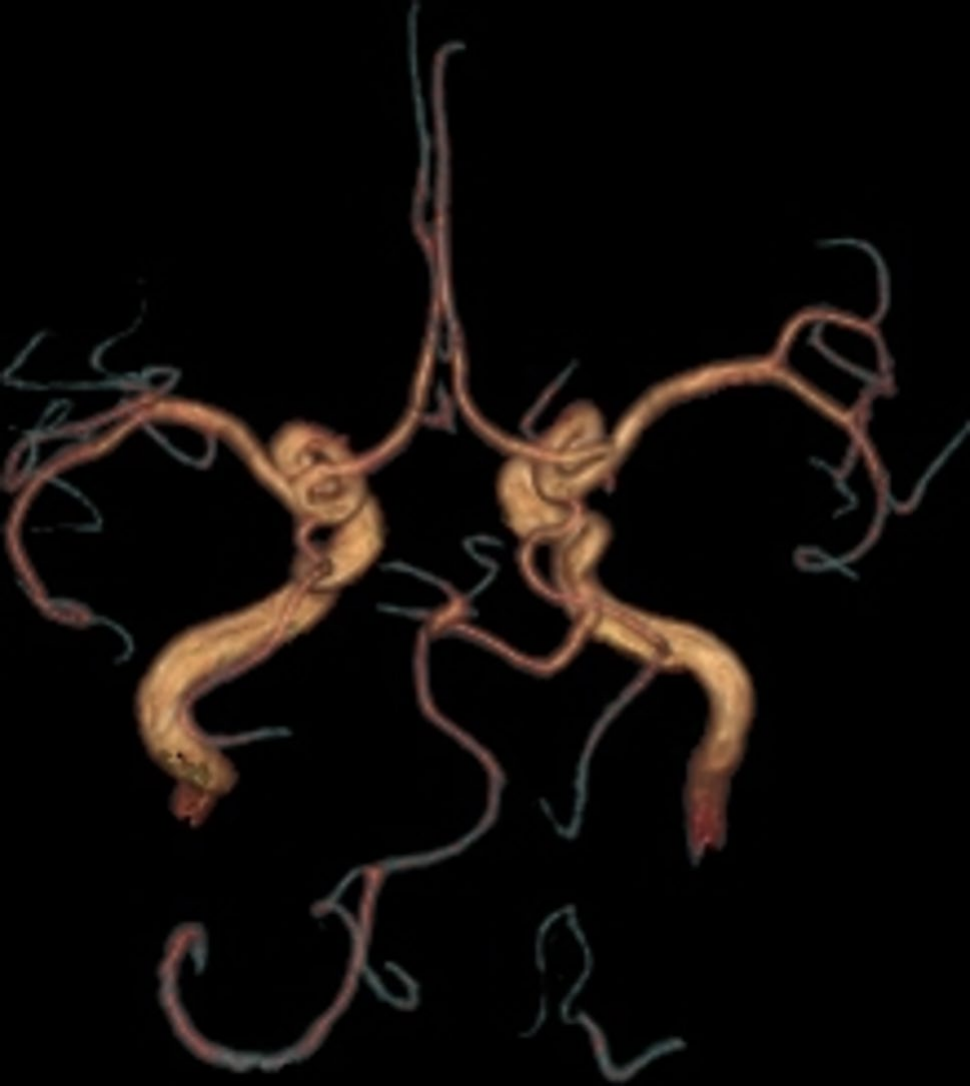
VR reconstruction of a TOF sequence, with a type II Saltzman trigeminal artery

This case, besides trigeminal artery, has other vascular anomalies: bilateral posterior cerebral artery P1 segment hypoplasia that has 1 mm diameter on the right 1,2 mm diameter on left, the flow in cerebral vascular territory beying assured by posterior communicating arteries, that has 1.6 mm diameter on the right, 1.8 mm on the left, so it can be included in Saltzman type II. The basilar artery is hypoplasic inferior to trigeminal artery anastomosis with a 1.4 mm diameter, and superior to this with a 2.8 caliber, similarly with trigeminal artery. Also has right vertebral artery hypoplasia and a median pericallosal artery, with origin from anterior communicating artery (Fig. 15).

Discussions.

In this study we found an 0.35% incidence for trigeminal artery, value that is similarly with [12, 15]. Although the study was made on few cases, only 3 trigeminal arteries, we observed a clear dominance for lateral type, described by Salas [16], situated lateral to sella turcica, with origin on internal carotid artery C4 segment superior or superolateral surface. The arteries traverse cavernous sinus where are in relation with oculomor, trochlear, abducens nerves and trigeminal nerve ophthalmic branch. They are exiting cavernous sinus, inferior to petroclinoid ligament entering in prepontine cistern where are joining basilar artery inferior to superior cerebellar arteries origin. Also we noticed woman dominance for this anatomical variant. Regarding the position to middle line we observed a right dominance with 2:1 ratio.

On the base of vascular territories, regarding [17] classification, we found equal proportion for type I and type II and one case was an intermediary between those two. If type I assures blood flow to cerebellum, brain stem and posterior inferior and medial part of cerebral hemispheres and type II assures blood flow only for cerebellum and brain stem, the second case described in this study, trigeminal artery assures blood flow for cerebellum, brain stem and only one brain hemisphere, variant that was not included in [17] classification.

In all cases there was a degree of basilar artery hypoplasia and hypoplasia or agenesis of vertebral arteries, most commonly the contralateral, in a ratio of 2:1. Those vascular anomalies lead to decreased blood flow to posterior cerebral fossa that is being supplied by internal carotid artery thru trigeminal artery. There has being described brain stem strokes in case of internal carotid artery or trigeminal artery occlusion in studies made by Kwon [10], Okada [11], Kato [9].

In other cases there have being reported symptoms like vertigo, nausea, vomiting, parenthesis, all specific for circulatory insufficiency in the vertebrobasilar system [18], similarly to the one reported in the first case. In their studies, Battista [4] and Eluvathingal [8] are supporting the theory of transient ischemic attacks, as cause of vertigo [1].

In all cases trigeminal artery was situated medial to trigeminal nerve and trigeminal nerve ophthalmic branch, running parallel with it. From a clinical point of view, trigeminal neuralgia is one of the most encountered pathologies associated with trigeminal artery [4]. In second case, trigeminal artery effectuates a lateral convex curvature, creating a vasculo nervous conflict with trigeminal nerve ophthalmic branch corresponding to same side hemicranias described by the patient.

In third case, trigeminal artery has a descending route out of the cavernous sinus and comes in contact with abducens nerve near Dorello channel, with evidence of external rectus muscle suffering. Other studies also described different degrees of ophthalmoplegia associated with trigeminal artery, depending on the trajectory and relation that this artery have and cranial nerves that are coming in contact with [6, 8].

In our study we haven’t found arterial aneurysm or arteriovenous malformations.

## Conclusions

Trigeminal artery is a rare anatomical variant, representing a embryonic vestige of the anastomosis between internal carotid artery and posterior circulator system, that can be asymptomatic or could have a vast clinical manifestations produce by flow insufficiency or by vasculo nervous conflicts.

Should not be forgotten, that is an important vessel situated in posterior cerebral fossa, which should be considered in neurosurgical intervention.

## Data Availability

The data presented in this study are available upon reasonable request to the corresponding author.
